# Investigating *Eucalyptus* – pathogen and pest interactions to dissect broad spectrum defense mechanisms

**DOI:** 10.1186/1753-6561-5-S7-P97

**Published:** 2011-09-13

**Authors:** Sanushka Naidoo, Ronishree Naidoo, Caryn Oates, Febe Wilken, Alexander Myburg

**Affiliations:** 1 FABI, University of Pretoria, South Africa

## Background

*Eucalyptus* species, hybrids and clones are attacked by various fungal and bacterial pathogens and pests during their life-time. Global climate changes are predicted to create favourable environments for such pathogens and pests and increase incidence of host jumping from other crops, resulting in increased losses to the forestry industry [1]. The use of tolerant or resistant plant varieties as part of an integrated disease management strategy is recognised as a desirable means to curb disease incidence. Vertical resistance mediated by resistance (*R*) genes, may be easily overcome by a pathogen and is thus not adequate on plantation species such as *Eucalyptus*, which would be exposed to various pathogens during its life-time. Broad spectrum resistance on the other hand, would be desirable to provide resistance against multiple challenges [2].

The aim of this study is to investigate mechanisms involved in host resistance with an emphasis on broad-spectrum resistance. The availability of the complete genome sequence of *Eucalyptus grandis* (http://www.eucagen.org) and the transcriptome sequence of a *E. grandis* X *E. urophylla* (GU) hybrid [3] has provided resources to investigate defense responses in the natural host. When a pathogen attacks a plant, the plant launches a sophisticated defense response involving phytohormones such as salicylic acid (SA), methyl jasmonate (MeJA) and ethylene (ET). These responses are finely tuned and tailored to the invader [4]. Down-stream of the signalling cascade is the production of pathogenesis related (*PR*) genes and antimicrobial genes which serve to limit the pathogen and afford protection. *PR* genes, such as *PR-1* and *PR-5,* are known markers of the salicylic acid defense pathway, while *PR-3*, *PR-4* and the lipoxygenase (*LOX*) genes are known markers of the MeJA and ET signalling pathways. The discovery of *PR* genes in *Eucalyptus* is desirable as these genes have previously been shown to afford broad spectrum resistance in other crops. We present our progress in exploiting the *Eucalyptus* genomic and transcriptomic data for the discovery of tree defense genes and explore the application thereof in determining which pathways are activated in response to various pathogens.

## Materials and methods

### Infections and infestations

*E. grandis* clones were treated with *Chrysoporthe austroafricana* in the following manner: wounds were created using a 0.3cm cork borer to expose the cambial tissue and an agar plug containing fungal mycelia were applied to the wound and sealed with parafilm. As a control, plants were mock inoculated. *E. nitens* plants were inoculated with *Phytophthora cinnamomi* using a 0.3cm cork borer, and a mycelial plug applied. The wound site containing the pathogen was sealed with moist cheese cloth and parafilm. A set of plants received no inoculumn. *E. grandis* clones were maintained in the FABI nursery and were naturally infested with *Leptocybe invasa*. A set of plants were maintained under similar conditions but were not exposed to the insect pest.

### Expression analysis

RNA was harvested from from stem tissue (from *C. austroafricana* and *P. cinnamomi* interactions) and leaf tissue (for *L. invasa* interactions) at two time points after challenge in order to detect early and late responses. RNA was isolated using the CTAB method [5] and cDNA synthesized. Reverse transcriptase quantitative PCR (RT-qPCR) was performed using the Roche 480 LightCycler instrument.

## Results and discussion

**Figure 1:**
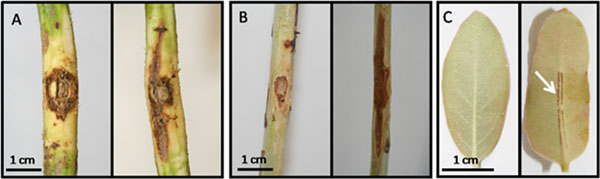
Results of infections and infestations on *Eucalyptus* species and clones. A) *E. nitens* challenged with *P. cinnamomi*, B) *E. grandis* clones challenged with *C. austroafricana* and C) *E. grandis* clones infested with *L. invasa*. Control, unchallenged plant material is indicated on the left and challenged plant material is indicated on the right of the respective panels.

Reliable pathosystems were established for *Eucalyptus* with *P. cinnamomi* and *C. austroafricana*. Figure 1A and 1B show the lesions which developed after pathogen challenge compared to mock inoculated plants. Figure 1C shows the results of natural infestation of young leaves of *E. grandis* clones under nursery conditions compared to plants not exposed to the pest. The oviposition sites are evident on the leaf midrib (indicated by the white arrow).

**Figure 2:**
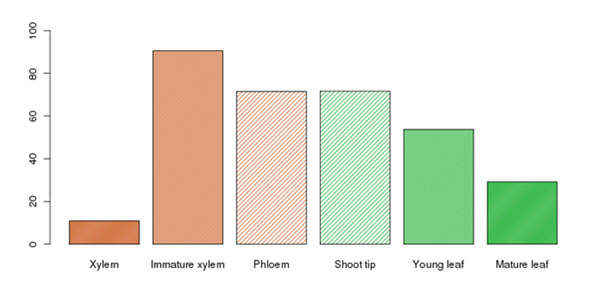
Relative expression levels of the putative *Eucalyptus PR-3* ortholog in various tissues of *E. grandis* x *E. urophylla* (data from Eucspresso [3]).

Using a bioinformatic and phylogenetic approach, the putative orthologs for *PR-1*, *PR-2*, *PR-3*, *PR-4* and *PR-5* were identified. The basal expression level of *PR-3* in the GU hybrid transcriptome is indicated in Figure 2 as an example.

The high basal expression of *PR-3* in the GU hybrid may indicate that the MeJA and ET pathway is activated in this genotype. *PR-3* genes are chitinases, enzymes that are able to hydrolyze chitin, a component of fungal cell walls [6]. Further expression profiling of the diagnostic marker genes for the two main defense pathways during *C. austroafricana* challenge suggests that the SA pathway is important for defense against the pathogen in the tolerant interaction.

## Conclusions

We have established important pathosystems between *Eucalyptus* and a fungal pathogen, *Eucalyptus* and an oomycete pathogen and between *Eucalyptus* and an insect pest. Genes diagnostic of the main defense signaling pathways have been identified and are being exploited to determine which pathways are activated in tree-pest/pathogen interactions. It is expected that transcriptome sequencing of each of these interactions will not only reveal the suite of genes important for defense against specific pathogens and pests, but the overlap of the responses at the molecular level, would be informative for broad spectrum resistance. These genes are potential future candidates for genetic improvement of disease tolerance in eucalypts.
